# The Biodiversity of Retreating Glaciers Leaves an Ecological Legacy in Emerging Soil Communities

**DOI:** 10.1111/gcb.71040

**Published:** 2026-08-14

**Authors:** Isabel Cantera, Silvio Marta, Alexis Carteron, Alessia Guerrieri, Simone Giachello, Aurélie Bonin, Roberto Ambrosini, Roberto Sergio Azzoni, Marco Caccianiga, Francesca Pittino, Andrea Simonicini, Barbara Valle, Mauro Gobbi, Gentile Francesco Ficetola

**Affiliations:** ^1^ Centre de Recherche en Biodiversité et Environnement (CRBE, UMR5300), CNRS, IRD, INP Université de Toulouse Toulouse France; ^2^ Department of Environmental Science and Policy University of Milan Milan Italy; ^3^ Institute of Geosciences and Earth Resources, CNR Pisa Italy; ^4^ Ecole d'Ingénieurs de Purpan, UMR INRAE‐INPT DYNAFOR University of Toulouse Toulouse France; ^5^ Argaly Montmélian France; ^6^ Department of Sciences, Technologies and Society University School for Advanced Studies IUSS Pavia Pavia Italy; ^7^ Department of Civil and Environmental Engineering São Paulo State University (UNESP) Bauru SP Brazil; ^8^ Centre of Applied Studies for the Sustainable Management and Protection of Mountain Areas (CRC Ge.S.Di.Mont.) University of Milan Edolo Italy; ^9^ Department of Earth Sciences “Ardito Desio” University of Milan Milan Italy; ^10^ Department of Biosciences University of Milan Milan Italy; ^11^ Department of Earth and Environmental Sciences (DISAT) University of Milano‐Bicocca Milan Italy; ^12^ Università degli Studi di Siena Siena Italy; ^13^ NBFC‐Nature Biodiversity Future Center Palermo Italy; ^14^ Research and Museum Collections Office, Climate and Ecology Unit MUSE‐Science Museum of Trento Trento Italy

**Keywords:** β‐diversity, ecological succession, glacier retreat, microbial traits, proglacial, supraglacial

## Abstract

Glacier retreat is transforming high‐mountain and polar landscapes, replacing ice with vast, newly exposed terrains where soil development and ecological succession start. Although ecosystems developing in deglaciated terrains are often viewed as starting from scratch, glaciers host diverse communities whose links with emerging soils remain unquantified. Using environmental DNA metabarcoding, we provide the first multi‐taxa assessment of biodiversity transitions across the supraglacial–proglacial interface across five glacier systems in Svalbard, the Alps and Patagonia. We analysed 212 samples from the surfaces of retreating glaciers and their forelands, spanning a chronosequence from 1 to 483 years since deglaciation. Recently deglaciated soils (≤ 10 years since glacier retreat) showed taxonomic and functional similarity to supraglacial communities. This ecological continuity was highly taxon‐specific, being pronounced for communities of microorganisms (Bacteria, Fungi, Protista) but weak for animals (e.g., Collembola, Insecta). For microbes, similarity diminished rapidly over succession as community turnover increased, and functional composition shifted from phototrophic and saprotrophic dominance toward heterotrophic and symbiotic assemblages. Shared microbial taxa and functions indicate a transient glacial influence during early soil succession. Our results suggest that early soil communities are not assembled independently of glacier ecosystems, but are initially linked to them through diverse ecological pathways that diminish over time. The observed glacier–proglacial connectivity challenges the view of succession on deglaciated terrains as beginning on lifeless substrates and instead points to a glacial legacy in emerging soils. As glaciers rapidly disappear under climate change, the unique biodiversity they host is also being lost, underscoring the urgent need to study supraglacial and proglacial systems together. Such integration is essential to understand ecological succession in proglacial ecosystems, which are expected to play an increasingly important role in this century.

## Graphical abstract

We provide the first multi‐taxa assessment of biodiversity transitions across the supraglacial‐proglacial interface, using eDNA metabarcoding across five glacier systems. Recently deglaciated soils resemble supraglacial communities taxonomically and functionally, markedly for microbes and weakly for animals, with microbial similarity declining rapidly as succession proceeds.

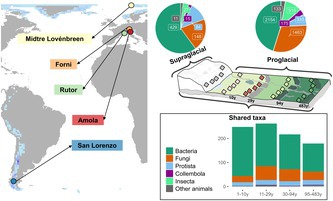

## 1 Introduction

Glaciers are retreating worldwide (Hugonnet et al. 2021; Zemp et al. 2025), exposing large barren terrains where new proglacial ecosystems emerge (Bosson et al. 2023; Ficetola et al. 2024). As climate change accelerates this retreat, these proglacial landscapes are projected to expand substantially (Bosson et al. 2023; Rounce et al. 2023; Zekollari et al. 2025; Zemp et al. 2025). Understanding their ecological dynamics is crucial to determine the fate of glacier‐associated biota (Gobbi et al. 2021) and how these landscapes will develop into diverse and productive ecosystems (Ficetola et al. 2021; Losapio et al. 2025; Zimmer et al. 2022).

Community assembly after glacier retreat begins with the biotic colonization of deglaciated terrains and proceeds through complex successional patterns that vary across environmental contexts and taxonomic groups (Ficetola et al. 2021, 2024; Guerrieri et al. 2024; Pothula and Adams 2022). Microbes (e.g., cyanobacteria, phototrophic protists) are typically the first colonizers, rapidly attaining high diversity and abundance (Bradley et al. 2014; Donhauser and Frey 2018; Praeg et al. 2025). They contribute to nutrient and organic matter accumulation, which, together with abiotic processes, supports the onset of soil development and the establishment of taxa requiring more time or less extreme conditions (Cauvy‐Fraunié and Dangles 2019; Ficetola et al. 2024; Giachello et al. 2024; Pothula and Adams 2022; Trejos‐Espeleta et al. 2024). Successional differences across taxa reflect variation in dispersal ability, tolerance to extreme abiotic conditions, and reliance on biotic interactions (Connell and Slatyer 1977; Ficetola et al. 2024; Pothula and Adams 2022). Despite growing interest in the successional trajectories of deglaciated terrains, their links with glacial biodiversity remain poorly understood (Stibal et al. 2020).

Ecological succession after glacier retreat has traditionally been viewed as primary succession on lifeless substrates (Ficetola et al. 2021), often overlooking the role of glacier ecosystems (Stibal et al. 2020). Yet, glaciers host active and diverse communities that influence biogeochemical cycles and sustain key linkages with surrounding ecosystems (Hågvar et al. 2024; Hågvar and Ohlson 2013; Stibal et al. 2020; Wojcik et al. 2021). Despite this, studies on post‐glacial succession have usually ignored their potential to supply nutrients, food, shelter, and propagules to emerging soil communities (Stibal et al. 2020), thereby influencing community assembly. Assessing how biodiversity and functions transition across the glacial–proglacial interface is therefore essential to understand community assembly and the factors shaping ecosystem development in deglaciated terrains.

The ecosystem at the surface of glaciers (Hodson et al. 2008) hosts structured communities, dominated by bacteria and fungi (Bradley et al. 2014; Crosta et al. 2024), and also supports an underappreciated diversity of animals such as tardigrades, rotifers, nematodes, and arthropods (Hågvar et al. 2020; Simoncini et al. 2026; Stibal et al. 2020; Zawierucha et al. 2015). These organisms can enter proglacial environments through active movement, passive transport by meltwater or katabatic wind, or by remaining embedded in glacial sediments released during ice melt (Hågvar et al. 2020, 2024; Rime et al. 2016; Tenan et al. 2016; Wojcik et al. 2021). Such biological input may contribute to the initial assembly of proglacial communities, thereby creating ecological linkages between glacial and proglacial ecosystems. For instance, bacteria detected in communities on proglacial lakes (Zhang et al. 2024) and recently deglaciated terrains (Rime et al. 2016) were suggested to originate from supraglacial habitats. For animals, the direction and ecological relevance of movements are more complex. Arthropods reported on both glacier surfaces and recently deglaciated terrains (≤ 10 years after deglaciation) are primarily proglacial species that use the ice temporarily as habitat or foraging ground, whereas ice specialists transported downslope rarely establish in proglacial habitats (Gobbi et al. 2017; Hågvar et al. 2024; Tenan et al. 2016). These complex, taxon‐specific exchanges highlight the importance of linkages between glacier and proglacial habitats. Such linkages may influence multiple features of recently deglaciated soils, such as nutrient availability, soil development and community composition, and would support the emerging view that succession in deglaciated terrains does not begin from scratch (Ficetola et al. 2021; Hågvar et al. 2024). However, the extent to which different components of glacier biodiversity contribute to soil communities, and how long they persist along successional gradients, remains unclear.

**FIGURE 1:**
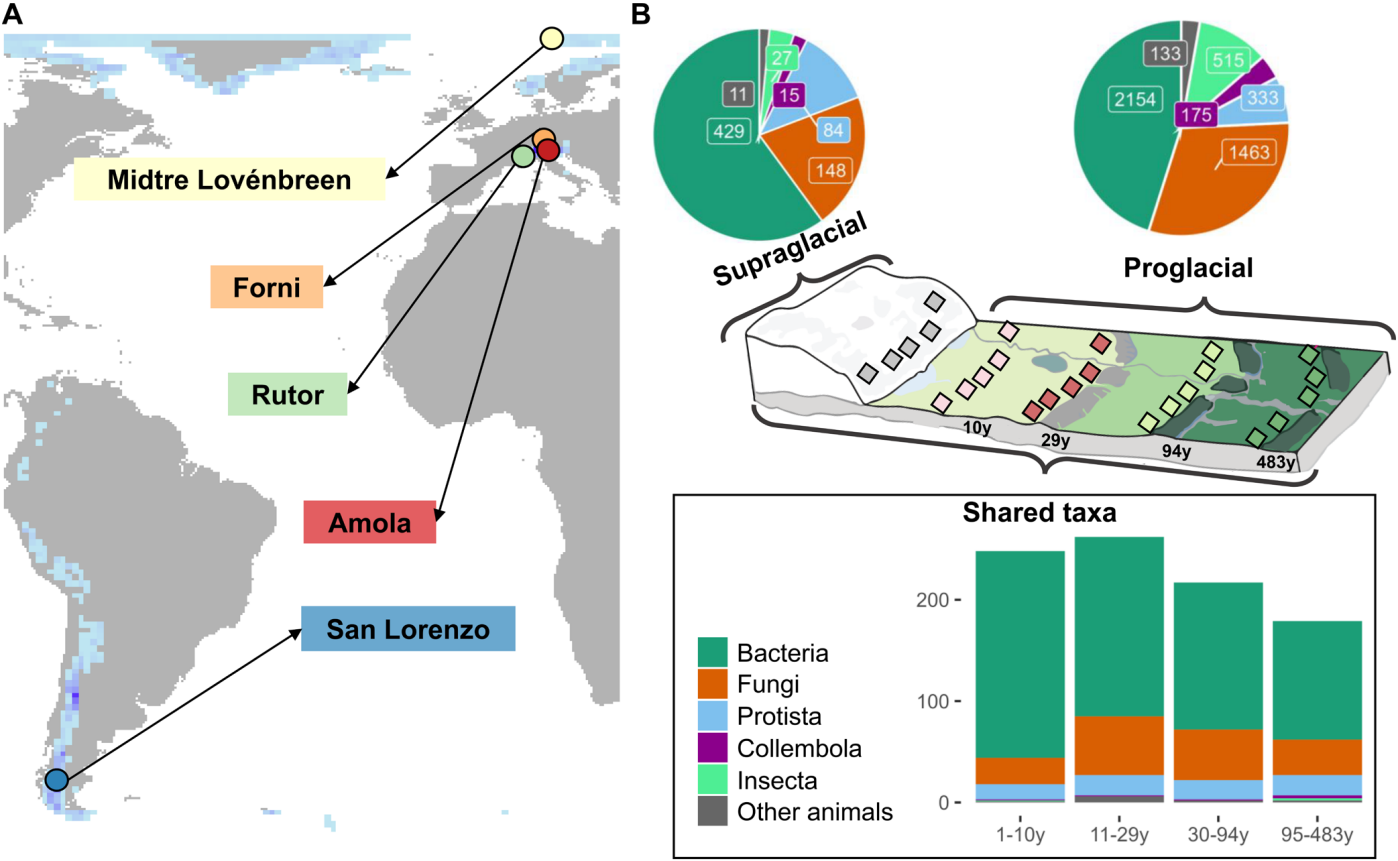
Study area, sampling design and detected taxa. (A) Location of the sampled retreating glaciers. (B) At each glacial landscape, we collected supraglacial samples and soil samples along the chronosequences of the glacier forelands, the latter representing different successional stages (1–483 years since deglaciation). Overall, we collected 25 supraglacial and 187 proglacial samples for environmental DNA metabarcoding analyses. Proglacial samples were grouped into four successional stages: recently deglaciated terrains (≤ 10 years after glacier retreat, *N* = 34 samples), soils in early (11–29 years, *N* = 49 samples), mid (30–94 years, *N* = 55 samples), and late successional stages (> 95 years, *N* = 49 samples). For each sample, six taxonomic groups were targeted. Pie charts show the proportion and number of taxonomic units for each group. Barplots represent the number of shared taxa between supraglacial and proglacial communities across successional stages.

To address this gap, we characterized communities inhabiting the surfaces of five retreating glaciers and the developing soils along their proglacial chronosequences (Figure 1) spanning 1–483 years since glacier retreat. The taxonomic and functional diversity of supraglacial communities was compared with that of proglacial communities at different successional stages, using environmental DNA (eDNA) metabarcoding to assess a large breadth of biodiversity, from microorganisms (bacteria, fungi, protists) to animals (Figure 1), with a consistent approach (Ficetola and Taberlet 2023). By characterizing supraglacial–proglacial transitions in biodiversity, we aimed to quantify ecological linkages between these ecosystems across taxa. If biological exchanges contribute to soil succession, we expect a continuum of similarity between supraglacial and proglacial communities that weakens from recently deglaciated terrains to late successional soils. Specifically, we hypothesize that: (1) community structure will shift through time, with recently deglaciated terrains hosting poor but functionally glacier‐like communities, and later stages becoming richer and more functionally distinct; and (2) the number of shared taxa between supraglacial and proglacial ecosystems will decline over time, implying an increasing taxonomic β‐diversity (particularly turnover) and decreasing nestedness along the successional gradient. Because glacier–foreland exchanges differ among taxa, we expect these patterns to be pronounced for microbes, which dominate supraglacial communities (Bradley et al. 2014) and can persist in early deglaciated soils (Rime et al. 2016), but weaker or blurred for animals, reflecting transient movements rather than establishment.

## 2 Materials and Methods

### 2.1 Sample Collection

We sampled supraglacial and proglacial communities (212 samples) in five retreating glaciers (Figure 1A) between 2016 and 2019 during the warmest months (i.e., mid‐summer, corresponding to the snow‐free period). Three glaciers were in the European Alps (Amola, Forni and Rutor), one in the Southern Andes (San Lorenzo) and one in the Arctic (Svalbard: Midtre Lovénbreen). Amola and Midtre Lovénbreen have forelands located above the treeline, while the forelands of Forni, Rutor, and San Lorenzo cross the treeline. For each glacier, communities were sampled by establishing plots on the surface of glaciers (supraglacial samples; Figure 1B) and plots on developing soils along glacier forelands (proglacial samples; Figure 1B), for a total of 25 supraglacial and 187 proglacial plots across all five glaciers (Table S1). Proglacial plots were regularly spaced (distance: ~20 m) within each of 44 dated sites. To cover different times since glacier retreat, we selected 5–17 dated sites (mean = 8.8) within each glacier foreland, corresponding to the position of the glacier front at a given time (Figure 1B), and thus creating a chronosequence (Walker et al. 2010). The number of sites and their age depended on accessibility and the availability of information on past glacier positions. Soil ages (time since glacier retreat) were determined from published reconstructions of glacier retreat based on historical archival records. A complete description of the approaches used for each foreland is available in Marta et al. (2021). At each site, we established 2–7 plots as distinct spatial replicates of the same age (see Table S1 for the number of plots per age for each glacier). Plots were selected to avoid areas affected by disturbances (erosion, streams, surface instability, grazing and heavy recreational activities), with superimposed deposits of different ages (e.g., outwash or landslides), and with uncertain dating. Proglacial samples spanned 1–483 years since deglaciation (Table S1). Throughout the text, we use the term “soil,” even though the collected samples represent very different stages of soil development, including non‐consolidated sediments (particularly immediately after glacier retreat), as well as the initial and intermediate stages of soil development (Khedim et al. 2021). For the supraglacial ecosystem, we established five plots above each glacier. Supraglacial plots were regularly spaced (distance: ~20 m) in areas of the glacier with the presence of supraglacial debris. All plots in the glacial landscape (both supraglacial and glacier foreland) were sampled during the same week.

At each plot, we collected five subsamples of soil or supraglacial debris within a 1‐m radius (see Ficetola et al. (2024), for details). The subsamples within each plot were pooled into a ~200 g composite sample (total of 212 composite samples; one distinct composite sample per plot/community), from which 15 g of soil/sediment was immediately desiccated in a sterile box with 40 g of silica gel to ensure the preservation of eDNA (Guerrieri et al. 2021). Disposable gloves, facemasks, and a portable blow torch (approximately 1000°C) were used for sample collection to prevent contamination.

### 2.2 eDNA‐Based Biodiversity Inventories

eDNA was extracted and amplified from composite soil and supraglacial debris samples following the protocol described in Guerrieri et al. (2023) and Ficetola et al. (2024), which has been widely applied in eDNA metabarcoding studies of glacier foreland soils (Cantera et al. 2024; Carteron et al. 2024; Giachello et al. 2024; Guerrieri et al. 2024). In brief, eDNA was extracted from the dried samples of each plot using the NucleoSpin Soil Mini Kit (Macherey‐Nagel) with one negative extraction control every approximately 23 samples. To provide a multi‐taxa assessment of the samples, five markers were used to amplify the extracted DNA. Two generalist primer pairs were used, targeting bacteria (Bact02) (Graça et al. 2024) and eukaryotes (Euka02, amplifying all Eukaryota) (Guardiola et al. 2015). In addition, we added three specific primer pairs: one targeting fungi (Fung02, amplifying Mycota) (Epp et al. 2012) and two targeting key soil animal groups: Insecta (Inse01) (Clarke et al. 2014; Graça et al. 2024) and Collembola (Coll01) (Janssen et al. 2018). All these markers have proved high performance for soil metabarcoding analyses (Calderón‐Sanou et al. 2024; Graça et al. 2024; Lunghi et al. 2022; Martinez‐Almoyna et al. 2019). Primers were tagged with 8‐nucleotide‐long tags on the 5′ end to allow bioinformatic discrimination of the four PCR replicates after sequencing (Coissac 2012). Extracted DNA was randomized in 96‐well plates with extraction controls, blanks, PCR‐negative and PCR‐positive controls (Zinger et al. 2019). See Ficetola et al. (2024) for details on controls used to evaluate cross‐contamination and to assess the performance of amplification and sequencing. The optimal number of amplification cycles was assessed using quantitative PCR and amplification was performed as previously described in Guerrieri et al. (2023). Libraries were prepared following the MetaFast protocol (Taberlet et al. 2018) and sequenced using the MiSeq (Bact02 and Fung02) or HiSeq 2500 (all others) Illumina platforms (Illumina; 2 × 250 bp for Bact02 and Fung02, and 2 × 150 bp for the other markers) at Fasteris (SA). The average sequencing depth was approximately 40,000 raw reads per sample for all the markers.

Bioinformatic treatment, filtering, clustering and taxonomic assignation were performed using the OBITools software suite (Boyer et al. 2016) and the sumaclust program (https://git.metabarcoding.org/obitools/sumaclust/wikis/home). We dereplicated sequences using the obiuniq program and discarded bad‐quality sequences, sequences shorter or longer than expected and singletons, as their removal is advised to increase the robustness of results (Alberdi et al. 2018; Brown et al. 2015). We then ran the obiclean program to detect potential PCR and sequencing errors, keeping sequences tagged as “heads” in at least one PCR replicate. Sequences were clustered into molecular operational taxonomic units using taxon‐specific similarity thresholds (Bonin et al. 2023). Subsequently, we performed an additional filtering to remove contaminants and spurious sequences (Calderón‐Sanou et al. 2020; Zinger et al. 2019). We discarded taxonomic units observed less than 12 (Euka02), 11 (Coll01), or 5 (Inse01, Fung02, and Bact02) times overall. These thresholds removed 99.99% or more of sequences detected in the blanks (i.e., tag‐jump errors) for each marker. We also discarded molecular operational taxonomic units detected in just one sample, as they often represent spurious sequences (Bálint et al. 2016), in less than two PCR replicates of the same sample (possible false positives, Ficetola et al. 2015) and in more than one extraction or PCR‐negative control (possible contaminants, Zinger et al. 2019). Additional details on bioinformatics and filtering are provided in Guerrieri et al. (2023). Several studies have shown that eDNA metabarcoding and traditional surveys provide highly consistent estimates of biodiversity (Ariza et al. 2023; Pansu et al. 2015). Moreover, previous comparisons across multiple forelands showed an excellent match between soil eDNA metabarcoding and traditional data for both plants and insects, supporting the robustness of eDNA‐based estimates of taxonomic richness (Ficetola et al. 2024) and β‐diversity (Cantera et al. 2024) in these systems.

### 2.3 Data Analysis

Given that the Euka02 primer can amplify eukaryotes that might also be amplified by the specific primers (Fung02, Inse01, and Coll01), we removed the sequences amplified by the Euka02 marker that were assigned to Fungi, Insecta, and Collembola. The removed sequences are indicated in Table S2. We did this to avoid duplicated detections of taxa that were not assigned at a fine taxonomic level with the Euka02 marker, given that specific markers have better taxonomic resolution (Ficetola and Taberlet 2023; Graça et al. 2024). We also removed taxa assigned to Streptophyta. The remaining sequences detected with the Euka02 marker were classified as either Protista (non‐fungal unicellular eukaryotes) or “other animals.” The group of other animals contained sequences assigned to Arthropoda (excluding Insecta and Collembola), Gastrotricha, Mollusca, Nematoda, Platyhelminthes, Annelida, Rotifera, and Tardigrada (Table S2). Moreover, one sequence assigned to Porifera was detected in only 2 samples with very few reads and a low match to known sequences of this phylum (88.8%, Table S2), suggesting misidentification due to database incompleteness; it was therefore excluded from all analyses.

In this study, “communities” refer to the assemblages detected in each sample within each taxonomic group. Communities in each sample were grouped into five ecosystem stages representing key stages of soil succession (Pothula and Adams 2022), while ensuring a balanced number of observations per stage (Figure 1B): supraglacial communities (*N =* 25 samples); recently deglaciated terrains (time since glacier retreat ≤ 10 years; *N* = 34 samples); early (11–29 years, *N* = 49 samples), mid (30–94 years, *N* = 55 samples), and late (> 95 years, *N* = 49 samples) successional stages. The first decade after glacier retreat was treated as a separate stage because it marks a transition from bare land to an environment able to support more complex communities, representing a critical period for nutrient accumulation, the establishment of microbial communities, and the initial, slow development of plants and animals (Ficetola et al. 2024; Pothula and Adams 2022). The subsequent stages were defined using quantile‐based binning of time since glacier retreat. This separation allowed for sequential comparisons along the successional gradient with comparable sample sizes between compared categories.

For each community, we calculated taxonomic richness using Hill's number Q1. This index corresponds to the exponential of Shannon entropy, balancing taxonomic richness and evenness by accounting for how evenly reads are distributed across taxonomic units, based on relative abundances. Hill's numbers were shown to provide more robust estimates of alpha‐diversity in metabarcoding studies compared to the number of detected taxonomic units (Calderón‐Sanou et al. 2020; Mächler et al. 2020). Read counts per taxon were log‐transformed to reduce the influence of highly amplified taxa. To test the effect of ecosystem stage (Figure 1B) on taxonomic richness, Q1 values were log‐transformed and fitted in linear mixed‐effects models (*lmer* function from *lme4* package), with glacier identity as a random intercept. Models were run separately for each taxonomic group. Significance of the ecosystem stage effect was assessed using type III ANOVA. When the overall ecosystem stage effect was significant, we performed sequential post hoc pairwise comparisons between stages using the *glht* function from the *multcomp* package. To assess gradual changes in taxonomic richness along the successional gradient, we compared only consecutive stages, treating successional stage as an ordinal factor: supraglacial versus recently deglaciated terrains (≤ 10 years since glacier retreat), recently deglaciated terrains versus early (11–29 years) successional communities, early versus mid (30–94 years) and mid versus late (95–483 years).

Trophic groups were assigned to each taxon corresponding to microorganisms (bacteria, protists and fungi), as they were the main biodiversity components of supraglacial samples (representing > 90% of all detected taxa). For fungi, trophic traits were assigned by extracting traits from FUNGuild (Nguyen et al. 2016). We kept only assignments classified as “highly probable” or “probable.” For bacteria and protists, traits were extracted from published databases (Calderón‐Sanou et al. 2024; Giachello et al. 2023, respectively) and trait assignment was done at the finest taxonomic level available for each taxon (see Table S2). Trophic category names are not directly equivalent across taxonomic groups, as they derive from different classification schemes. We were able to assign a trophic group to all protist taxonomic units, and to 64% of bacterial and 63% of fungal taxonomic units (Table S2). To identify shared trophic traits between supraglacial and proglacial communities over succession, we assessed the relationships between trophic categories and ecosystem stages using the fourth‐corner statistic (Dray and Legendre 2008) (*fourthcorner* function of the *ade4* package; Dray and Dufour 2007). This analysis assessed the link between relative abundances of detected taxa per sample, the ecosystem stage where the sample was collected, and the trophic traits associated with each taxon. A permutation model combining permutations of entire rows (communities) and columns (taxa) (i.e., the sixth permutation model) was used to assess significant relationships by mitigating the risk of Type I errors (Dray and Legendre 2008; Ter Braak et al. 2012). The *p*‐values were corrected to control for errors associated with testing multiple hypotheses simultaneously, using the False Discovery Rate (FDR) procedure, which controls the expected proportion of false rejections of the null hypothesis among all rejections (Benjamini and Hochberg 1995; Benjamini and Yekutieli 2001). To account for spatial autocorrelation when assessing trophic–environment relationships, we applied Moran's Spectral Randomization to the fourth‐corner analysis using the *msr* function from the *adespatial* package (Braga et al. 2018). Spatial weights derived from the *dnearneigh* function from the *spdep* package were used to account for the spatial relationships between sampling communities from the same glacial landscape (i.e., 5 clusters) based on geographic distances. We conducted the fourth‐corner analyses for each taxonomic group separately, with 9999 permutations.

To assess continuity across the glacier–proglacial interface, we calculated taxonomic dissimilarity (total β‐diversity) between supraglacial and proglacial communities differing in time since glacier retreat (Figure 1B). Within each glacial landscape, only supraglacial–proglacial pairs were retained, excluding supraglacial–supraglacial and proglacial–proglacial comparisons to avoid conflating ecologically distinct processes. High β‐diversity indicates low similarity between supraglacial and proglacial communities. For each taxonomic group, β‐diversity was computed from presence/absence matrices using Jaccard's index. We tested whether β‐diversity between supraglacial and proglacial communities was related to the age of the proglacial community to which the supraglacial diversity was compared. To this aim we used matrix regression with block permutation following the framework of multiple regression on distance matrices (Lichstein 2007), which handles the non‐independence of pairwise distances (i.e., the same sample contributes to multiple pairs). Supraglacial samples were assigned a time value of zero, so the pairwise time distance between each supraglacial–proglacial pair is equivalent to the time since deglaciation of the proglacial sample involved in the comparison. Geographic distance was not included as an additional predictor because time and geographic distance matrices showed high correlation (*r* = 0.70), and our primary interest concerns successional rather than spatial patterns. Pairwise β‐diversity was modelled as a function of pairwise differences in time using a linear mixed model (*lmer*, *lme4* package), with glacier identity included as a random intercept to account for baseline differences in dissimilarity among glacial landscapes. Due to the non‐independence of pairwise distances, significance was then assessed through block permutation (1000 permutations), in which rows and columns of the response matrix were permuted independently within each glacier block and only the intra‐glacier supraglacial–proglacial pairs were retained. This allowed preserving the within‐glacier structure of dissimilarities while generating a null distribution against which the observed relationship between β‐diversity and time since deglaciation was assessed. *p*‐values were calculated as the frequency of permuted coefficients with absolute values larger than the absolute observed coefficient, after correcting them for the explained variance (pseudo‐*t*‐test; Lichstein 2007). Conditional *R* ^2^ was extracted using the *r.squaredGLMM* function from the *MuMIn* package. Models were run separately for each taxonomic group. The number of intra‐glacier supraglacial–proglacial pairs included in each model varied across taxonomic groups due to the exclusion of pairs involving sites with no detected taxa, for which Jaccard dissimilarity is undefined: Bacteria = 935 pairs, Fungi = 928, Insecta = 900, Protista = 879, Other animals = 813, and Collembola = 796.

To distinguish compositional turnover from richness differences, β‐diversity was partitioned into turnover and nestedness components following Baselga (2010) and using the *betapart* package (Baselga and Orme 2012). Nestedness measures the extent to which one community is a strict subset of another, with higher values indicating a higher proportion of shared taxa (overlap) between supraglacial and proglacial communities. Turnover quantifies compositional replacement independently of richness, isolating pure compositional shifts across succession (Baselga 2010). For each taxonomic group and successional stage, we pooled all pairwise comparisons and calculated the percentage of turnover and nestedness explaining total β‐diversity within each successional stage. Finally, to evaluate whether recently deglaciated soils (≤ 10 years) were compositionally closer to supraglacial communities than to soils at later successional stages, we compared the turnover between recently deglaciated terrains and supraglacial samples against the turnover between recently deglaciated terrains and each later successional stage (early: 11–29 years; mid: 30–94 years; late: > 95 years) using pairwise Wilcoxon tests with Benjamini–Hochberg correction for multiple comparisons.

## 3 Results

### 3.1 Taxonomic Shifts From Supraglacial Habitats to Developing Soils

After clustering and removal of spurious or not targeted sequences, eDNA metabarcoding recovered 4953 taxonomic units across all samples, with 714 detected in supraglacial samples and 4773 in proglacial samples (Table S2). Overall, bacteria showed the highest number of taxonomic units in both ecosystems, followed by fungi (Figure 1B). In supraglacial communities, bacteria were the dominant group (> 60% of detected taxonomic units, Figure 1B), and this dominance was consistent across all glaciers (Figure S1). Fungi and protists were also present in notable amounts in supraglacial communities, and their relative proportion varied among glaciers (Figure S1). In contrast, proglacial communities exhibited an increase in the relative proportion of eukaryotic taxa (fungi, protists, and animals) (Figure 1B). Protists were a particularly important component in supraglacial samples, while they accounted for a lower proportion of taxa in proglacial soils, where eukaryotic dominance was mainly driven by fungi and animals rather than protists (Figure 1B). Insects were rare in supraglacial communities and accounted for a higher proportion of taxa in proglacial soils (Figure 1B). Taxonomic units assigned to springtails and other animals represented < 4% of all taxa detected in both ecosystems (Figure 1B). Besides insects and springtails, several animal taxonomic units corresponding to mites, nematodes, tardigrades, and rotifers were detected in supraglacial samples (Table S2).

**FIGURE 2:**
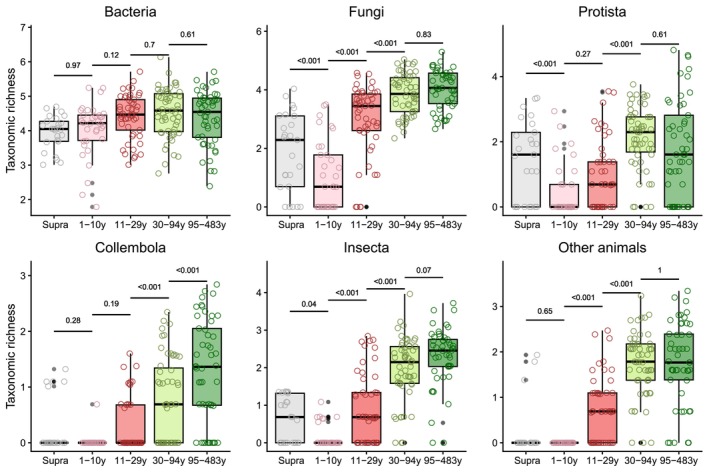
Changes in taxonomic richness from supraglacial to soil ecosystems. Taxonomic richness was quantified across ecosystem stages: Supraglacial samples (“Supra”), recently deglaciated terrains (≤ 10 years after glacier retreat), soils in early (11–29 years), mid (30–94 years), and late (> 95 years) successional stages along glacier chronosequences. Boxplots show the distribution of richness values for each group and indicate median (middle line), 25th and 75th percentiles (box), as well as ranges of 1.5 × interquartile range (whiskers) and outliers (dots). *p*‐values from sequential post hoc pairwise comparisons between successive groups (based on mixed linear models) are displayed. Points represent each sampled community.

The total number of taxonomic units per sample ranged from 24 to 147 in supraglacial samples (mean = 85, SE = 6.6) and from 8 to 587 in proglacial samples (mean = 174, SE = 8.1). Taxonomic richness (Hill's number *Q* = 1) varied significantly among ecosystem stages (ANOVA: *F*_4,186–206_ ≥ 4.1, *p* ≤ 0.003 for all groups, Table S3, Figure 2). For most groups, taxonomic richness in supraglacial communities exceeded that of recently deglaciated terrains (≤ 10 years), except for bacteria (Figure 2). This initial decrease was significant for fungi, protists and insects (sequential pairwise comparisons between consecutive stages: all *p* ≤ 0.04, Figure 2). Subsequently, taxonomic richness significantly increased with time since glacier retreat for all groups except bacteria—earlier (recently deglaciated to early‐successional stages) for fungi, insects and other animals, and later (early to mid‐successional stages) for protists and springtails (sequential pairwise comparisons: all *p* < 0.001) (Figure 2).

### 3.2 Functional Shifts From Supraglacial Habitats to Developing Soils

**FIGURE 3:**
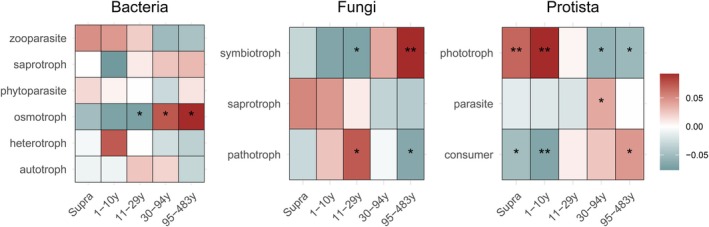
Functional shifts from supraglacial to soil ecosystems. Heatmaps represent the relationships between trophic groups and ecosystem stages for microorganisms across the 212 samples. The relative abundances of taxa, ecosystem stages, and trophic traits were linked through the fourth‐corner analysis. Colors represent the D2 statistic, indicating the strength and direction of the relationship: Red (positive association), white (neutral), and blue (negative association). Asterisks denote statistical significance of the association: *p ≤ 0.05; **p ≤ 0.01 after adjusting for multiple comparisons using the false discovery rate procedure.

To assess shifts in community functional composition, we applied the fourth‐corner analysis to identify trophic groups associated with the different ecosystem stages (Figure 3). We focused on bacteria, fungi, and protists, as animal diversity was low in supraglacial samples, with some samples containing no detected animals (Table S2). Phototrophic protists were more frequent than expected in supraglacial habitats and terrains ≤ 10 years old; then their relative abundance declined significantly after ~30 years since deglaciation. Conversely, consumer protists were underrepresented in supraglacial and recently deglaciated communities and significantly associated with the latest successional stage. Parasitic protists showed a significant positive association with mid‐successional soils (30–94 years), but no clear association with other stages. For bacteria and fungi, fewer significant associations were detected. Among bacteria, osmotrophs were less frequent in supraglacial samples and soils younger than 30 years, while they increased significantly in later stages (Figure 3). No fungal group was specifically associated with the supraglacial ecosystem, but symbiotrophs tended to be less frequent in supraglacial samples and soils younger than 30 years, and more frequent in later stages (Figure 3).

### 3.3 Similarity Between Supraglacial and Proglacial Communities Over Succession

**FIGURE 4:**
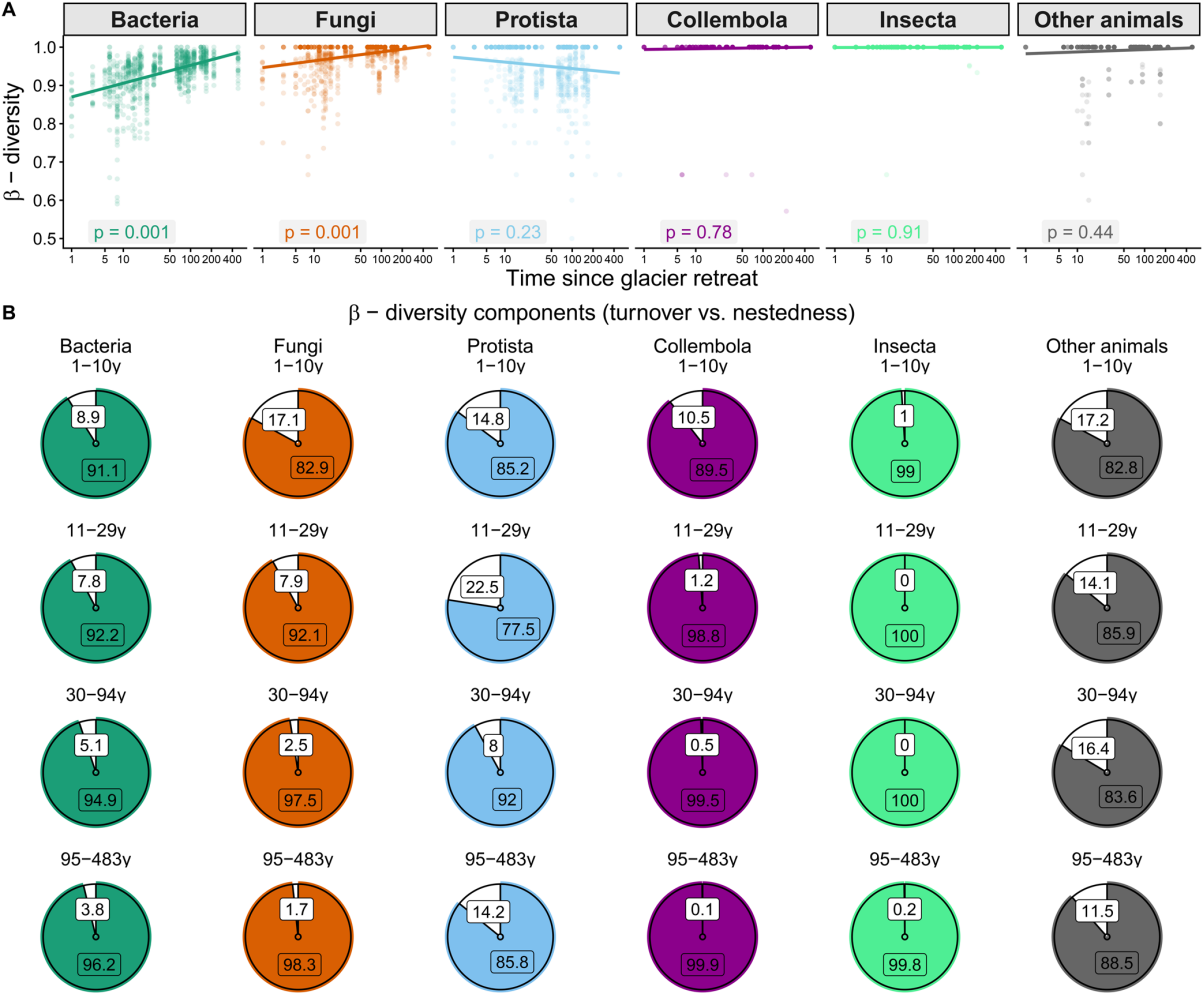
Similarity between supraglacial and proglacial communities over succession. (A) Relationships between time since glacier retreat and the total β‐diversity (community dissimilarity) between supraglacial (*N* = 25 samples) and proglacial communities (*N* = 187 samples). Plots show regression lines, with *p*‐values obtained via block permutation and pseudo‐*t* test. (B) Relative contributions of turnover (colored) and nestedness (white) to total β‐diversity between supraglacial and proglacial communities across successional stages. For each stage and group, we calculated the average percentage of dissimilarity explained by each component across samples. Soil communities were sampled in recently deglaciated, early‐, mid‐, and late‐successional stages along glacier chronosequences.

For each glacier landscape, we compared the communities on glacier surfaces with those in their respective proglacial forelands to assess taxonomic continuity across the glacier–proglacial interface. Overall, β‐diversity (dissimilarity) between supraglacial and proglacial communities was high for all groups (β‐diversity > 0.5, Figure 4A), indicating strong compositional differences within each glacier landscape. Nonetheless, bacteria, fungi, and protists exhibited comparatively lower dissimilarities than animals and a higher proportion of shared taxa between supraglacial and proglacial communities (Figure 1B). For bacteria and fungi, β‐diversity increased with time since deglaciation (Bacteria: *R* ^2^_c_ = 0.46, slope = 0.00026, *p* = 0.001; Fungi: *R* ^2^_c_ = 0.277, slope = 0.000092, *p* = 0.001, Figure 4A, Table S4), suggesting a progressive differentiation of communities as succession proceeds. No temporal trend was detected for protists and animals (all *p* ≥ 0.2, Table S4); the latter likely due to their low richness in supraglacial samples.

Overall, β‐diversity was mostly explained by turnover, while nestedness contributed modestly (Figure 4B). Across glaciers, 402 taxa were shared between supraglacial and proglacial communities (Table S2), mostly microbes (Figure 1B). Bacteria showed the highest percentage of shared taxonomic units (11.9% of all bacteria), followed by protists (10.6%) and fungi (5.5%). The quantification of the relative contributions of nestedness and turnover to β‐diversity between supraglacial and proglacial communities revealed that the proportion of shared taxa varied across groups and successional stages (Figure 4B). Nestedness between supraglacial communities and communities in recently deglaciated soils (≤ 10 years) ranged from 9% to 17% in bacteria, fungi and springtails, but declined to < 4% in late‐successional stages. Other groups showed no temporal pattern across the successional gradient (Figure 4B).

Finally, across most taxonomic groups, turnover between supraglacial samples and recently deglaciated soils (≤ 10 years) was lower than turnover between recently deglaciated soils and more mature successional stages (> 30 years). For bacteria, fungi, springtails, and other animals, turnover between supraglacial and recently deglaciated communities was significantly lower than turnover between recently deglaciated and mid‐successional soils (pairwise Wilcoxon tests: *p* < 0.01, Figure S2, Table S5); and also significantly lower than turnover with the late‐successional stage for bacteria, fungi, and springtails, but not for other animals. Differences between supraglacial–recently deglaciated turnover and recently deglaciated–early successional (11–29 years) turnover were generally weaker or not significant, except for fungi, which showed a significantly higher turnover between recently deglaciated soils and supraglacial samples than between recently deglaciated soils and early successional soils. In contrast, protists and insects showed no significant differences among ecosystem stages (Figure S2, Table S5).

## 4 Discussion

Post‐glacial ecosystems are expected to have an increasingly important role in the next century (Bosson et al. 2023). Identifying the processes promoting early succession in these ecosystems is fundamental for understanding and predicting the ecological changes currently occurring in rapidly warming high‐latitude and high‐altitude ecosystems. Our study provides the first multi‐taxa assessment of ecological connectivity between glacier surfaces and their forelands, revealing community‐level linkages across the supraglacial–proglacial interface that are quantifiable and vary among taxonomic groups and over time. For some taxa, communities on recently deglaciated terrains (≤ 10 years after glacier retreat) are more similar to the supraglacial ones than to soil communities established later in succession (Figure S2). Succession in emerging post‐glacial ecosystems is thus shaped by complex and dynamic processes reflecting both environmental modifications and the traits of colonizing organisms.

The detected supraglacial communities were dominated by microorganisms, encompassing all major trophic groups (Figure S3). Their composition aligns with previous findings. Phototrophic bacteria and protists thrive in the light‐abundant, nutrient‐poor conditions of supraglacial habitats, driving primary production (Anesio et al. 2017; Stibal et al. 2012; Zhang et al. 2024). Saprotrophic fungi are also prevalent, contributing to organic matter decomposition and nutrient cycling (Crosta et al. 2024; Franzetti et al. 2020). This trophic structure was mirrored in recently deglaciated terrains (≤ 10 years, Figure 3), where the same groups prevailed but taxonomic richness was generally lower than on supraglacial communities. The initial years following glacier retreat are critical for the build‐up of nutrient pools (Hågvar et al. 2020), during which phototrophic microbes and saprotrophic fungi act as keystone taxa driving autochthonous primary production and nutrient accumulation (Bradley et al. 2014). The growing availability of nutrients and organic matter can subsequently promote the establishment of other organisms (Yu et al. 2025). As soil develops and ecological interactions increase in complexity (van Leeuwen et al. 2018), the role of these pioneers diminishes, giving way to symbiotic fungi, osmotrophic bacteria, parasites and consumer protists that exploit organic compounds accumulating in the soil. This functional shift—from producer‐dominated microorganisms in both supraglacial and pioneer ecosystems to more complex communities hosting more heterotrophs in later stages—parallels vegetation development, marked by increasing richness and competition over time (Cantera et al. 2024; Ficetola et al. 2024). The transition from sparse vegetation to grasslands and forests reduces light and resources for autotrophic and saprotrophic microorganisms, whereas heterotrophs, mutualists, and parasites benefit from the growing presence of organisms (e.g., animals) and resource availability (Carteron et al. 2024). The continuity in trophic structure across the supraglacial–proglacial interface suggests that, for micro‐organisms, communities in recently deglaciated terrains are not randomly assembled but are closely linked to supraglacial ones, likely reflecting both the persistence of taxa with traits adapted to cold, low‐nutrient conditions and ecological similarities near the ice margin, where shared environmental filters may shape community structure.

Supraglacial and proglacial communities shared several taxa, predominantly microbes (Figure 1B), with bacteria and fungi showing the clearest patterns of overlap and directional change. Indeed, β‐diversity between supraglacial and proglacial communities increased significantly along the successional gradient, while nestedness declined rapidly (Figure 4). The presence of shared taxa aligns with the trophic continuity across the supraglacial–proglacial interface, highlighting detectable taxonomic and functional cross‐ecosystem linkages. Recently deglaciated terrains are fleeting ecosystems that receive inputs from the glacier; supraglacial materials and organic matter can remain in place after glacier melt, contributing to sediment formation in newly deglaciated environments, thereby triggering early succession (Bardgett et al. 2007; Guelland et al. 2013; Hågvar and Ohlson 2013; Wojcik et al. 2021). In this context, the observed microbial similarity suggests that supraglacial diversity might also influence early proglacial communities, as suggested by other studies (Rime et al. 2016; Zhang et al. 2024). Such contributions can involve functional roles that trigger fundamental early ecological processes like primary production and nutrient cycling, prior to plant establishment and before other heterotrophic and symbiotic taxa become dominant. This functional shift converges with changes in carbon sources (Hågvar and Ohlson 2013), as heterotrophic microbes initially exploit allochthonous glacial carbon and switch to local plant‐derived carbon after ~50 years (Guelland et al. 2013; Vinšová et al. 2022). These patterns suggest that early soil development depends partly on glacial legacies that support early biodiversity and energy flow, reinforcing the view that succession in deglaciated terrains does not begin from a sterile state (Ficetola et al. 2021; Hågvar et al. 2024), but is influenced in its earliest stages by glacial ecosystems. However, this influence appears short‐lived: ecological continuity between supraglacial and proglacial communities diminishes as community turnover intensifies over succession (Cantera et al. 2024; del Moral 2009; Hanusch et al. 2022; Yu et al. 2025). Early similarities likely reflect both biological transfer from glacier surfaces and shared environmental constraints, but these effects weaken as succession progresses and new environmental filters emerge. Such rapid changes are consistent with the inherently dynamic nature of succession (Cantera et al. 2024), where stochastic and allogenic drivers dominate early stages before being overtaken by biotic interactions and autogenic feedbacks (Wojcik et al. 2021).

Patterns of supraglacial–proglacial similarity varied markedly among taxonomic groups. In contrast to microbes, sequences of animals were scarce on glacier surfaces, and very few taxa were shared across ecosystems, suggesting a limited ecological continuity that may arise from transient exchanges (both active and passive) rather than established populations. Some groups (e.g., tardigrades, rotifers, springtails) include species that depend on glacial habitats, some of which are glacier specialists exclusively found in glacial habitats (Simoncini et al. 2026). The number of taxonomic units attributed to these animals was generally low (see Table S2), and such low values were expected considering the limited species richness typically observed in supraglacial environments (Crosta et al. 2024; Simoncini et al. 2026). For instance, traditional approaches reported that just one tardigrade and a few rotifer species dominate the supraglacial environment of the Forni Glacier (Crosta et al. 2024; Zawierucha et al. 2019). Isotomid springtails were detected on both supraglacial surfaces and soils younger than 10 years (Figure 4B), consistent with their known ecology on the ice and at glacier–foreland interfaces (Valle, Barbon, et al. 2025; Valle, D'Adda, et al. 2025; Valle et al. 2021), where they can feed on cyanobacteria, diatoms, and mosses (Hågvar et al. 2024). Other animals can temporarily occupy ice margins or young soils for foraging or dispersal, including ground beetles and spiders (Simoncini et al. 2026; Tenan et al. 2016). Furthermore, several arthropods found on glaciers likely represent wind‐transported taxa rather than resident taxa (Hågvar et al. 2020). For example, we found several sequences of aphids on supraglacial samples, which are plant‐feeding insects that do not inhabit glacier surfaces but likely arrive as aeroplankton (Hågvar et al. 2020). Thus, the animals detected at the earliest stages include both cryophilic specialists (Hågvar and Ohlson 2013) and transient organisms that, even without persisting, may contribute to the establishment of food webs as sources of organic matter supporting higher trophic levels (Crosta et al. 2024; Hågvar 2012; Hågvar et al. 2020, 2024; Hågvar and Gobbi 2022; Hågvar and Ohlson 2013; Hodkinson et al. 2002; Schmidt et al. 2014). The contrasting patterns of microorganisms and animals highlight the multiple pathways, including the supply of nutrients, food, shelter, and/or propagules (Stibal et al. 2020), through which biodiversity connects glacial and proglacial habitats during early succession.

Ecological connectivity between supraglacial and proglacial habitats was detectable but weak, with low nestedness relative to turnover, suggesting limited overlap between ecosystems. Supraglacial habitats are highly selective, shaped by low temperatures, intense solar radiation, and limited nutrients (Zhang et al. 2024), allowing only a few specialists to persist. In contrast, the greater environmental heterogeneity of glacier forelands provides a broader range of niches and receives colonizers from diverse neighboring ecosystems such as grasslands, mountain slopes, and mature forests, leading to greater diversity and community exchange (Ficetola et al. 2021; Wojcik et al. 2021). This diversification increases local richness but also marks a transition from cold‐adapted specialists to more generalist assemblages later in succession (Cauvy‐Fraunié and Dangles 2019). Some glacier‐associated taxa may persist briefly thanks to the microhabitat similarity between supraglacial and recently deglaciated terrains (i.e., cold temperatures, prolonged snow cover, water persistence after snow melt and nutrient scarcity, Ficetola et al. 2021; Hågvar et al. 2020; Losapio et al. 2025; Marta et al. 2023), but this is short‐lived. An integrative view of ecological changes in glaciated landscapes as dynamic mosaics is needed, as the disappearance of cryospheric habitats will profoundly reshape dispersal pathways, nutrient fluxes, and biodiversity patterns of surrounding ecosystems in a warming world (Losapio et al. 2025; Milner et al. 2017).

Because eDNA can persist after an organism's death, sequences detected in recently deglaciated soils could, in principle, include DNA from species no longer present. However, studies show that eDNA quickly decreases after the removal of the organisms, that topsoil eDNA mostly represents organisms present on site within recent years (Ariza et al. 2023; Bithell et al. 2015; Foucher et al. 2020; Guthrie et al. 2024), and that eDNA‐based estimates of soil biodiversity strongly match those from traditional approaches (Ariza et al. 2023; Cantera et al. 2024; Ficetola et al. 2024). This is related to the quick degradation of eDNA molecules under aerobic, wet conditions (Taberlet et al. 2018; Turner et al. 2015). Although some DNA from non‐resident organisms may persist, such sporadic detection is unlikely to bias our overall community pattern, particularly given the broad temporal span of the successional stages considered here. eDNA metabarcoding nonetheless provides here an integrated signal of ecological connectivity between glacial and proglacial ecosystems, reflecting both biological exchanges and shared abiotic constraints near the ice margin. Clarifying the direction and mechanisms of this connectivity for each taxonomic group will require combining morphological, physiological and environmental data to separate true colonization from passive dispersal and to identify the relative roles of biotic and abiotic supraglacial inputs in early soil assembly.

Our findings address long‐standing gaps in understanding ecological succession following glacier retreat (Ficetola et al. 2021; Stibal et al. 2020; Wojcik et al. 2021). Rather than being an independent process occurring on lifeless ground, succession in deglaciated terrains begins within a brief phase in which forelands are connected to their glaciers. Through dispersal, persistence, and shared environmental filters, glacier‐related organisms can momentarily contribute to primary production, nutrient cycling, and energy flow in newly exposed soils. This transient process leaves a measurable taxonomic and functional imprint, forming a glacial legacy that shapes early soil development, showing that glacier ecosystems are not isolated end‐members, but interactive components of mountain landscapes. With global warming, glacier habitats, and the unique biodiversity they host, are disappearing or projected to disappear within decades (Huss et al. 2017; Linsbauer et al. 2025; López‐Moreno et al. 2025; Losapio et al. 2025; Valle, D'Adda, et al. 2025; Veettil and Kamp 2019). Given this, recognizing glaciers as dynamic nodes linked to their proglacial landscapes through living, non‐living, and short exchanges is essential to predict the trajectories of deglaciated areas and uncover the functional legacy of glaciers (Losapio et al. 2025).

## Author Contributions

**Alessia Guerrieri:** writing – review and editing, methodology, formal analysis, data curation. **Aurélie Bonin:** writing – review and editing, methodology, data curation, formal analysis. **Mauro Gobbi:** writing – review and editing, validation, investigation, resources. **Simone Giachello:** writing – review and editing, data curation, methodology. **Marco Caccianiga:** writing – review and editing, investigation. **Silvio Marta:** writing – review and editing, data curation, formal analysis, methodology. **Roberto Sergio Azzoni:** writing – review and editing, resources. **Alexis Carteron:** writing – review and editing, data curation. **Andrea Simonicini:** writing – review and editing. **Barbara Valle:** writing – review and editing, investigation, resources. **Isabel Cantera:** conceptualization, investigation, writing – original draft, methodology, validation, visualization, formal analysis. **Gentile Francesco Ficetola:** conceptualization, investigation, funding acquisition, writing – original draft, methodology, validation, project administration, supervision, resources. **Francesca Pittino:** writing – review and editing, resources. **Roberto Ambrosini:** writing – review and editing, investigation, resources.

## Funding

This work was supported by European Research Council (772284) and Biodiversa+ (101052342).

## Conflicts of Interest

The authors declare no conflicts of interest.

## Supporting information


**Table S1:** Information on the collected samples on five retreating glaciers (*N* = 212). In each glacier, we collected environmental DNA from 5 supraglacial samples and 25–65 soil samples along the glacier forelands. Overall, we collected 25 supraglacial samples and 187 proglacial samples along the chronosequences of the glacier forelands representing different successional stages (1–483 years since deglaciation). For each sample, we targeted 6 taxonomic groups. For each sample, the table indicates the code of the glacier and its complete name, the plot identifier within each site, the geographical locations where the samples were collected, the sampling year, the time since glacier retreat (years), the minimum distance from the glacier front (in meters, for supraglacial samples only), the environment from which the sample was collected (supraglacial or proglacial), the ecosystem type and the taxonomic richness calculated for each taxonomic group, expressed as both Hill's number Q1 and raw taxonomic richness (Q0). Ecosystem stages were defined as follows: supraglacial communities (“Supra,” *N* = 25 samples); recently deglaciated terrains (≤ 10 years since glacier retreat, *N* = 34); early succession (11–29 years, *N* = 49); mid succession (30–94 years, *N* = 55); and late succession (> 95 years, *N* = 49).
**Table S2:** Taxonomic units per sample (*N* = 212) in supraglacial and proglacial ecosystems across five retreating glaciers. The table indicates whether each sequence was found in supraglacial, proglacial, or both ecosystems (“Shared”). The table further includes the marker used to amplify the taxonomic unit, its corresponding sequence, best identity thresholds, the assigned trophic groups for microbes (Bacteria, Fungi and Protista), taxonomic assignments, and the total number of reads across four PCR replicates after bioinformatic filtering. Taxonomic units removed to avoid redundant detections between the Euka02 marker and specific markers are also noted (see Section 2).
**Table S3:** Results of ANOVA tests applied to linear mixed‐effects models assessing the effect of ecosystem stage on taxonomic richness (Hill number, *Q* = 1) for each taxonomic group, with ecosystem stage included as a fixed effect and glacier identity as a random intercept. The table reports *F*‐values and *p*‐values obtained from type III ANOVA. Ecosystem stages were defined as follows: supraglacial communities (“Supra,” *N* = 25 samples); recently deglaciated terrains (≤ 10 years since glacier retreat, *N* = 34); early succession (11–29 years, *N* = 49); mid succession (30–94 years, *N* = 55); and late succession (> 95 years, *N* = 49).
**Table S4:** Results of matrix regression with block permutation (1000 permutations) testing whether β‐diversity between supraglacial and proglacial communities is related with time since glacier retreat, for each taxonomic group. Models were fitted on intra‐glacier supraglacial–proglacial pairs only, with supraglacial samples assigned a time value of zero such that pairwise time distances correspond to the time since glacier retreat of the proglacial sample. Glacier identity was included as a random intercept to account for baseline differences in dissimilarity among glaciers. Significance of the time coefficient was assessed via block permutation rather than the model's parametric *p*‐values, since pairwise distances violate the independence assumptions of standard mixed‐model inference. Conditional *R* ^2^, reflecting variance explained by both the fixed effect (time) and the glacier random intercept, is reported. The number of intra‐glacier supraglacial–proglacial pairs included in each model varied across taxonomic groups due to the exclusion of pairs involving sites with no detected taxa, for which Jaccard dissimilarity is undefined: Bacteria = 935, Fungi = 928, Insecta = 900, Protista = 879, Other animals = 813, and Collembola = 796.
**Table S5:** Pairwise Wilcoxon test statistics comparing turnover values computed between recently deglaciated soils and supraglacial samples (Supra versus 1–10 years comparisons), against turnover values computed between recently deglaciated soils and each other successional stage (1–10 years vs. 11–29 years, 1–10 years vs. 30–94 years, and 1–10 years vs. 95–483 years comparisons), for each taxonomic group. *n*1 and *n*2 indicate the number of pairwise sample comparisons contributing to each compared group; *p* and *p*.adj indicate raw and Benjamini–Hochberg‐adjusted *p*‐values, respectively.
**Figure S1:** Number of detected taxonomic units in supraglacial and proglacial communities per each taxonomic group in each glacier (slore = San Lorenzo and midtr = Midtre Lovénbreen).
**Figure S2:** Comparison of turnover values between recently deglaciated soils (≤ 10 years after glacier retreat) and other ecosystem stages: supraglacial (“Supra”) samples, early (11–29 years), mid (30–94 years), and late (> 95 years) successional soils. Turnover captures differences in species composition independently of species richness, isolating pure compositional shifts across succession. Boxplots display the distribution of turnover values for each group, showing the median (line), interquartile range (box), 1.5× interquartile range (whiskers), and outliers (points). *p*‐values are from pairwise Wilcoxon tests with Benjamini–Hochberg correction, comparing the turnover between recently deglaciated soils and supraglacial samples against the turnover between recently deglaciated soils and each later successional stage (early, mid, late), to test whether turnover differs between the immediate post‐glacial transition and later successional stages.
**Figure S3:** Proportion of taxa detected for each trophic group in supra‐and proglacial samples.

## Data Availability

The generation and processing of this dataset are fully described in Guerrieri et al. (2023) and Ficetola et al. (2024). Raw sequence data and filtered sequence data are publicly available in Zenodo repositories (https://doi.org/10.5281/zenodo.7628236 and https://doi.org/10.5281/zenodo.10423968, respectively) (Carteron 2024; Guerrieri et al. 2022). Formatted data that support the findings of this study are provided as Tables S1 and S2. The scripts for reproducing the results in this study are available as Codes S1 and S2.
