# Supplementary material for: The Biodiversity of Retreating Glaciers Leaves an Ecological Legacy in Emerging Soil Communities
Source: Glob Chang Biol. 2026 Aug 14;32(8):e71040. doi: 10.1111/gcb.71040 (PMC13473894; doi:10.1111/gcb.71040)
Supplement: Supplementary file 1 [file GCB-32-e71040-s001.zip › gcb71040-sup-0001-TableS1-S5-FigureS1-S3@cantera_2026_Supplementary materials (1).docx]

# Supplementary materials for

**The biodiversity of retreating glaciers leaves an ecological legacy in emerging soil communities**

Isabel Cantera^*1,2^, Silvio Marta^3^, Alexis Carteron^4^, Alessia Guerrieri^5^, Simone Giachello^2,6^, Aurélie Bonin^5^, Roberto Ambrosini^2,7,8^, Roberto Sergio Azzoni^2,9^, Marco Caccianiga^10^, Francesca Pittino^11^, Andrea Simonicini^2^, Barbara Valle^12,13^, Mauro Gobbi^14^ and Gentile Francesco Ficetola^2^

^1^Centre de Recherche en Biodiversité et Environnement (CRBE, UMR5300), CNRS, IRD, INP, Université de Toulouse, Toulouse, France.

^2^Department of Environmental Science and Policy, University of Milan, Milan, Italy.

^3^Institute of Geosciences and Earth Resources, CNR, Pisa, Italy.

^4^University of Toulouse, Ecole d’Ingénieurs de Purpan, UMR INRAE-INPT DYNAFOR, Toulouse, France.

^5^Argaly, 1 Place du Centenaire, 73800, Montmélian, France.

^6^ Department of Sciences, Technologies and Society, University School for Advanced Studies IUSS Pavia, Pavia, Italy.

^7^Department of Civil and Environmental Engineering, São Paulo State University (UNESP), Av. Eng. Luis Edmundo C. Coube, n. 14-01 Bauru-SP – BRAZIL

^8^Centre of Applied Studies for the Sustainable Management and Protection of Mountain Areas (CRC Ge.S.Di.Mont.), University of Milan, Via Morino 8, 25048, Edolo, Italy

^9^Department of Earth Sciences “Ardito Desio”, University of Milan, Milan, Italy

^10^Department of Biosciences, University of Milan, Milan, Italy.

^11^Department of Earth and Environmental Sciences (DISAT) - University of Milano-Bicocca, Milano, Italy.

^12^Università degli Studi di Siena, Siena, Italy.

^13^NBFC- Nature Biodiversity Future Center, Palermo, Italy.

^14^Research and Museum Collections Office, Climate and Ecology Unit, MUSE-Science Museum of Trento, Italy.

*Corresponding author: isabel.cantera@ird.fr
**Supplementary material includes:**

Supplementary Tables 1 to 5

Supplementary Figures 1 to 3

# Supplementary tables

## Table S1. Information on the collected samples on five retreating glaciers (N = 212). On each glacier, we collected environmental DNA from 5 supraglacial samples and 25-65 soil samples along the glacier forelands. Overall, we collected 25 supraglacial samples and 187 proglacial samples along the chronosequences of the glacier forelands representing different successional stages (1-483 years since deglaciation). For each sample, we targeted 6 taxonomic groups.

## The table indicates the code of the glacier and its complete name, the plot identifier within each site, the geographical locations where the samples were collected, the sampling year, the time since glacier retreat (years), the minimum distance from the glacier front (in meters, for supraglacial samples only), the environment from which the sample was collected (supraglacial or proglacial), the ecosystem type and the taxonomic richness calculated for each taxonomic group, expressed as both Hill's number Q1 and raw taxonomic richness (Q0). Ecosystem stages were defined as follows: supraglacial communities ("Supra", N = 25 samples); recently deglaciated terrains (≤10 years since glacier retreat, N = 34); early successional (11–29 years, N = 49); mid successional (30–94 years, N = 55); and late successional (>95 years, N = 49) stages.

## Table S2. Taxonomic units per sample (N = 212) in supraglacial and proglacial ecosystems across five retreating glaciers. The table indicates whether each sequence was found in supraglacial, proglacial, or both ecosystems (“Shared”).

## The table further includes the marker used to amplify the taxonomic unit, its corresponding sequence, best identity thresholds, the assigned trophic groups for microbes (Bacteria, Fungi and Protista), taxonomic assignments, and the total number of reads across four PCR replicates after bioinformatic filtering. Taxonomic units removed to avoid redundant detections between the Euka02 marker and specific markers are also noted (see Methods).

## Table S3. Results of ANOVA tests applied to linear mixed-effects models assessing the effect of ecosystem stage on taxonomic richness (Hill number, Q = 1) for each taxonomic group, with ecosystem stage included as a fixed effect and glacier identity as a random intercept. The table reports F-values and p-values obtained from type III ANOVA. Ecosystem stages were defined as follows: supraglacial communities ("Supra", N = 25 samples); recently deglaciated terrains (≤10 years since glacier retreat, N = 34); early succession (11–29 years, N = 49); mid succession (30–94 years, N = 55); and late succession (>95 years, N = 49).

| Taxonomic group | df (Num, Den) | F-values | *p*-value |
| --- | --- | --- | --- |
| Bacteria | (4, 204.2) | 4.1 | 0.003 |
| Fungi | (4, 203.6) | 73 | <0.001 |
| Protista | (4, 204.5) | 18.8 | <0.001 |
| Collembola | (4, 205.9) | 27.3 | <0.001 |
| Insecta | (4, 204.3) | 54.3 | <0.001 |
| Other animals | (4, 186) | 58.2 | <0.001 |

## Table S4. Results of matrix regression with block permutation (1000 permutations) testing whether β-diversity between supraglacial and proglacial communities is related to time since glacier retreat, for each taxonomic group. Models were fitted on intra-glacier supraglacial–proglacial pairs only, with supraglacial samples assigned a time value of zero so that pairwise time distances correspond to the time since glacier retreat of the proglacial sample. Glacier identity was included as a random intercept to account for baseline differences in dissimilarity among glaciers. Significance of the time coefficient was assessed via block permutation rather than the model's parametric p-values, since pairwise distances violate the independence assumptions of standard mixed-model inference. Conditional R², reflecting variance explained by both the fixed effect (time) and the glacier random intercept, is reported. The number of intra-glacier supraglacial–proglacial pairs included in each model varied across taxonomic groups due to the exclusion of pairs involving sites with no detected taxa, for which Jaccard dissimilarity is undefined: Bacteria = 935, Fungi = 928, Insecta = 900, Protista = 879, Other animals = 813 and Collembola = 796.

| Taxonomic_group | Conditional R***²*** | Time coefficient | Time *p*-value |
| --- | --- | --- | --- |
| Bacteria | 0.461 | 0.000263 | 0.001 |
| Fungi | 0.277 | 0.000092 | 0.001 |
| Protista | 0.049 | 0 | 0.228 |
| Collembola | 0.008 | 0 | 0.784 |
| Insecta | 0.005 | 0 | 0.909 |
| Other animals | 0.094 | 0 | 0.44 |

**Table S5.** Pairwise Wilcoxon test statistics comparing turnover values computed between recently deglaciated soils and supraglacial samples (Supra *versus* 1-10y comparisons), against turnover values computed between recently deglaciated soils and each other successional stage (1-10y *versus* 11-29y, 1-10y *versus* 30-94y, and 1-10y *versus* 95-483y comparisons), for each taxonomic group. n1 and n2 indicate the number of pairwise sample comparisons contributing to each compared group; *p* and *p*.adj indicate raw and Benjamini–Hochberg-adjusted *p*-values, respectively.

| **Taxonomic group** |  | **Group1** | **Group2** | **n1** | **n2** | **W** | ***p*** | ***p*.adj** |
| --- | --- | --- | --- | --- | --- | --- | --- | --- |
| Bacteria |  | Supra_1-10y | 1-10y_11-29y | 170 | 476 | 44169 | 0.076 | 0.076 |
| Bacteria |  | Supra_1-10y | 1-10y_30-94y | 170 | 420 | 13928.5 | **<0.001** | **<0.001** |
| Bacteria |  | Supra_1-10y | 1-10y_95-483y | 170 | 270 | 6074.5 | **<0.001** | **<0.001** |
| Fungi |  | Supra_1-10y | 1-10y_11-29y | 115 | 364 | 23803 | **0.024** | **0.029** |
| Fungi |  | Supra_1-10y | 1-10y_30-94y | 115 | 315 | 15688.5 | **0.029** | **0.029** |
| Fungi |  | Supra_1-10y | 1-10y_95-483y | 115 | 205 | 9135.5 | **0.0005** | **0.001** |
| Protista |  | Supra_1-10y | 1-10y_11-29y | 54 | 162 | 4283 | 0.806 | 0.97 |
| Protista |  | Supra_1-10y | 1-10y_30-94y | 54 | 175 | 4626.5 | 0.808 | 0.97 |
| Protista |  | Supra_1-10y | 1-10y_95-483y | 54 | 98 | 2917 | 0.278 | 0.556 |
| Collembola |  | Supra_1-10y | 1-10y_11-29y | 61 | 20 | 681.5 | **0.0621** | **0.0621** |
| Collembola |  | Supra_1-10y | 1-10y_30-94y | 112 | 20 | 1288 | **<0.001** | **0.0001** |
| Collembola |  | Supra_1-10y | 1-10y_95-483y | 72 | 20 | 828 | **0.0009** | **0.0014** |
| Insecta |  | Supra_1-10y | 1-10y_11-29y | 67 | 201 | 7065 | 0.118 | 0.236 |
| Insecta |  | Supra_1-10y | 1-10y_30-94y | 67 | 255 | 8547.5 | 0.975 | 0.975 |
| Insecta |  | Supra_1-10y | 1-10y_95-483y | 67 | 165 | 5577.5 | 0.671 | 0.805 |
| Others animals |  | Supra_1-10y | 1-10y_11-29y | 5 | 73 | 161 | 0.387 | 0.464 |
| Others animals |  | Supra_1-10y | 1-10y_30-94y | 5 | 85 | 170 | <0.001 | 0.0003 |
| Others animals |  | Supra_1-10y | 1-10y_95-483y | 5 | 58 | 126 | 0.315 | 0.464 |

# Supplementary figures

**Figure S1.** Number of detected taxonomic units in supraglacial and proglacial communities per each taxonomic group in each glacier (slore = San Lorenzo and midtr = Midtre Lovénbreen).

### **Figure S2.** Comparison of turnover values between recently deglaciated soils (≤10 years after glacier retreat) and other ecosystem stages: supraglacial (‘'Supra’) samples, early (11–29 years), mid (30–94 years), and late (>95 years) successional soils. Turnover captures differences in species composition independently of species richness, isolating pure compositional shifts across succession. Boxplots display the distribution of turnover values for each group, showing the median (line), interquartile range (box), 1.5× interquartile range (whiskers), and outliers (points). *p*-values are from pairwise Wilcoxon tests with Benjamini–Hochberg correction, comparing the turnover between recently deglaciated soils and supraglacial samples against the turnover between recently deglaciated soils and each later successional stage (early, mid, late), to test whether turnover differs between the immediate post-glacial transition and later successional stages. Diamonds indicate means of turnover for each compared stage.

**Figure S3**. Proportion of taxa detected for each trophic group in supra-and proglacial samples.
